# Distillation of Regional Activity Reveals Hidden Content of Neural Information in Visual Processing

**DOI:** 10.3389/fnhum.2021.777464

**Published:** 2021-11-26

**Authors:** Trung Quang Pham, Shota Nishiyama, Norihiro Sadato, Junichi Chikazoe

**Affiliations:** ^1^Section of Brain Function Information, Supportive Center for Brain Research, National Institute for Physiological Sciences, Okazaki, Japan; ^2^Aichi Institute of Technology Graduate School of Business Administration and Computer Science, Toyota, Japan; ^3^Araya Inc., Tokyo, Japan; ^4^Division of Cerebral Integration, National Institute for Physiological Sciences, Okazaki, Japan

**Keywords:** MVPA, decoding, machine learning, fMRI, visions

## Abstract

Multivoxel pattern analysis (MVPA) has become a standard tool for decoding mental states from brain activity patterns. Recent studies have demonstrated that MVPA can be applied to decode activity patterns of a certain region from those of the other regions. By applying a similar region-to-region decoding technique, we examined whether the information represented in the visual areas can be explained by those represented in the other visual areas. We first predicted the brain activity patterns of an area on the visual pathway from the others, then subtracted the predicted patterns from their originals. Subsequently, the visual features were derived from these residuals. During the visual perception task, the elimination of the top-down signals enhanced the simple visual features represented in the early visual cortices. By contrast, the elimination of the bottom-up signals enhanced the complex visual features represented in the higher visual cortices. The directions of such modulation effects varied across visual perception/imagery tasks, indicating that the information flow across the visual cortices is dynamically altered, reflecting the contents of visual processing. These results demonstrated that the distillation approach is a useful tool to estimate the hidden content of information conveyed across brain regions.

## 1. Introduction

Brain decoding has drawn interest from neuroscientists for decades. Decoding gives meaning to the activity patterns inside the brain, thus providing a potential for reverse engineering in order to understand how the brain organizes and stores information. Recent studies have broadly utilized the multi-voxel pattern analysis (MVPA) of functional magnetic resonance imaging (fMRI) images as a standard tool to decipher what people are seeing (Haxby et al., 2001; Kamitani and Tong, 2005; Horikawa and Kamitani, 2017), hearing (Hoefle et al., 2018), imagining (Stokes et al., 2009; Reddy et al., 2010; Cichy et al., 2012), and dreaming (Horikawa et al., 2013).

In terms of targeted perception, vision has been the preferred candidate due to its simplicity. Visual processing, particularly visual object recognition, is a well-established hierarchical organization in both anatomical and functional aspects (Felleman and Van Essen, 1991). A recent study (Horikawa and Kamitani, 2017) presented a decoding approach for generic decoding of visual features in both perception and imagery tasks. The authors suggested that the mental imagery is a type of top-down processing, whereas mental perception is a bottom-up process. Interplay between top-down and bottom-up processing helps sharpen the neural representation of stimuli (Abdelhack and Kamitani, 2018). However, the top-down signals also cause bias in the early visual-sensitive area (Kok et al., 2012, 2013). Therefore, to unveil the “true pattern” reflecting the received visual stimuli, one should eliminate the influence of top-down signals.

In this study, MVPA of fMRI images was used to distill the unsullied pattern of activity in a region of interest (ROI). We assume the prediction of a low-level ROI based on the activity of a high-level ROI to specifically represent its top-down signals from that specific one, and the prediction of a high-level ROI based on the activity of a low-level signals to represent bottom-up signals. Using the open-access data obtained from (Horikawa and Kamitani, 2017), we demonstrated a region-to-region decoding technique in which the top-down/bottom-up signals at an ROI (target) are linearly integrated from the activity of the other regions (seeds). Thereafter, we examined the prediction of visual features of observed stimuli before and after eliminating the top-down/bottom-up signals during the perception and imagery tasks. Finally, we compared the magnitude of distillation effects between all possible seed–target pairs associated with the visual processing.

## 2. Materials and Methods

### 2.1. Data and Preprocessing

We used the preprocessed task fMRI data of 5 subjects in the publicly accessible Generic Object Decoding dataset (https://github.com/KamitaniLab/GenericObjectDecoding). This dataset was used to replicate Horikawa et al.' paper (Horikawa and Kamitani, 2017). MRI data were collected using 3.0-Tesla Siemens MAGNETOM Trio A Tim scanner from the ATR Brain Activity Imaging Center. An interleaved T2*-weighted gradient-echo plannar imaging (EPI) scan was performed [repetition time (TR), 3,000 ms; echo time (TE), 30 ms; flip angle, 80 deg; field of view [FOV], 192 × 192 *mm*^2^]. T1-weighted magnetization-prepared rapid acquisition gradient-echo fine-structural images of the entire head were also acquired (TR, 2,250 ms; TE, 3.06 ms; TI, 900 ms; flip angle, 9 deg, FOV, 256 × 256 *mm*^2^; voxel size, 1.0 × 1.0 × 1.0 *mm*^3^.

The fMRI data underwent three-dimensional motion correction using the SPM5 software (http://www.fil.ion.ucl.ac.uk/spm). Data were then coregistered with the whole-head high-resolution anatomical images. The coregistered data were then reinterpolated using 3 × 3 × 3 *mm*^3^ voxels. After within-run linear trend removal, voxel amplitudes were normalized relative to the mean activity of the entire time course within each run. To estimate the brain activity associated with each trial, the normalized voxel activity was then averaged within each 9-s stimulus block (image presentation experiment) or within each 15-s imagery period (imagery experiment), after shifting the delay the data by 3 s to compensate for hemodynamic delays.

This dataset consists of 1,200 training, 1,750 test (perception), and 500 test (imagery) trials for each subject. Visual images were collected from the online image database ImageNet (Deng et al., 2009). Two hundred representative object categories were selected as stimuli in the visual presentation experiment. In the training image session, a total of 1,200 images from 150 object categories (eight images from each category) were presented only once. In the test image session, a total of 50 images from 50 object categories (one image from each category) were presented 35 times each. Care was taken to avoid misuse of the categories for the test session during the training session. In the imagery experiment, the subjects were asked to visually imagine images from one of the 50 categories that were presented in the test image session of the image presentation experiment.

### 2.2. Region-to-Region Decoding

To estimate the information flow from a region to a region, we calculated the fine-grained topographic connectivity between regions (Heinzle et al., 2011). In this analysis, a single voxel activity in the target region was modeled by a weighted linear summation of all the voxel activities in the seed region. Considering that the activity of voxels in the same ROI is highly correlated, a ridge regression analysis was employed for weight estimation, but not ordinary least squares analysis. The ridge parameter was optimized such that the prediction performance in the validation dataset is maximized. For this purpose, we divided the training dataset into 600 training and 600 validation trials. In test dataset, the voxel activity in the target region was predicted through the estimated weights computed using the optimal ridge parameter.

To evaluate the region-to-region decoding performance, the average coefficients of determination (*R*^2^) among all the voxels in the target ROI were calculated. For comparison, functional connectivity was also calculated between regions. As the present dataset reflects task-related activity, the method proposed by Rissman et al. (2004) was used.

### 2.3. Bottom-Up/Top-Down Signal Elimination

We hypothesize that the observed activity of visual cortices reflects both bottom-up and top-down signals conveyed between the visual pathways. The bottom-up/top-down signals can be approximated using linear predictions through the observation of other ROIs. Prediction of a targeted ROI derived from a seed ROI can be expressed as follows.


$$X_{seed\to target}\approx a\times X_{seed}^{*}+b$$

where **a** and **b** denote the parameters of the linear regression. The $X_{seed}^{*}$ denotes the observed representation of a signal at the seed ROI. Then, the representation of the signal at the target ROI can be expressed as follows


$$X_{target}^{*}=X_{seed\to target}+X_{latent\_factor}$$

where $X_{target}^{*}$ denotes the observed representation of the signal at the target ROI, and the **X**_*latent*_*factor*_ represents “hidden” content at the target ROI which was subsequently used for visual feature prediction.

These expressions suggest that the activity of the primary visual cortex would directly reflect retinal input if the top-down signal from the higher visual cortex (such as the fusiform face area, FFA) could be appropriately eliminated. Considering this, the activity explained by the seed region (e.g., FFA) was eliminated from the target region (e.g., V1). The former activity is estimated using the region-to-region decoding technique described above.

### 2.4. Visual Feature Prediction

We tested 13 candidates of visual features, including a convolutional neural network (CNN1–CNN8) (Krizhevsky et al., 2012), HMAX model (HMAX1–HMAX3) (Riesenhuber and Poggio, 1999; Serre et al., 2007; Mutch and Lowe, 2008), GIST (Oliva and Torralba, 2001), and scale-invariant feature transform (SIFT) (Lowe, 1999) in combination with the bag of features (BOF) (Csurka et al., 2004). All visual features are continuous data. Among these, the multi-layered models (CNN and HMAX) represent the hierarchical processing of human visual systems. GIST provides a low-dimensional representation of a scene, specified for scene recognition. SIFT + BOF is similar to GIST but is designed for object recognition.

Visual feature vectors of seen objects were predicted from the activity patterns of each ROI, based on a linear regression function. To build the prediction model, we used the code available on Horikawa et al.'s website (https://github.com/KamitaniLab/GenericObjectDecoding). The sparse linear regression (SLR; http://www.cns.atr.jp/cbi/sparse_estimation/index.html) (Bishop, 2006) was used for automatically selecting the important features for prediction. The regression function can be expressed as follows:


$$y(x)=\sum_{i=1}^{d}w_{i}x_{i}+w_{0}$$

where *x*_*i*_ denotes the scalar value of the voxel *i*, *w*_*i*_ denotes the weight of voxel *i*, *w*_0_ denotes the bias, and *d* denotes the number of voxels in an fMRI sample **x**. For weight estimation, we adopted the variational Bayesian automatic relevance determination model (Sato, 2001; Tipping, 2001; Horikawa et al., 2013).

Hence, the weights of the regression function can be estimated by evaluating the following joint posterior probability of **w**:


$$P(w,\alpha ,\beta |X,t_{l})=\frac{P(t_{l}|X,w,\beta )P_{0}(w|\alpha )P_{0}(\alpha )P_{0}(\beta )}{\int dwd\alpha d\beta P(t_{l},w,\alpha ,\beta |X)}$$

where ***t***_***l***_ denotes the target variable of the *l*^*th*^ component of a visual feature explained by the *y*(**x**) with additive Gaussian noise; **w**, the weight vector of regression function; **α**, the weight precision parameters; and β, the noise precision parameter. The learning algorithm involves the maximization of the product of the marginal likelihood and the prior probabilities of **w**, **α**, and β.

We trained linear regression models that predicted the feature vectors of the individual feature types/layers for seen objects of the fMRI samples during the training session. For the test dataset, fMRI samples corresponding to the same categories (35 samples in the test image session and 10 samples in the imagery session) were averaged across trials to increase the signal-to-noise ratio of the fMRI signals. Using the trained models, feature vectors of seen/imagined objects from averaged fMRI samples were predicted to construct one predicted feature vector for each test category. Model fitting and prediction were conducted for each feature unit. A total of 100 feature units were randomly selected for each visual feature. As a metric of decoding accuracy, we calculated the correlation coefficient between true and predicted feature values of the 50 test images.

Correlation coefficients of 100 units from five participants were calculated, providing 100 × 5 correlation coefficients in each feature type/layer for each ROI. To evaluate the effect of the bottom-up/top-down signal elimination, the prediction modes were built before and after the signal elimination. The significance of the signal elimination effect was examined using a paired *t*-test. The correlation coefficient was preferred because we focused on feature decoding where the pattern across feature units is more important than the absolute value of a single unit. The mean absolute error (MAE) and mean squared error (MSE) were additionally measured for validating the findings derived from correlation analysis.

**Figure 1:**
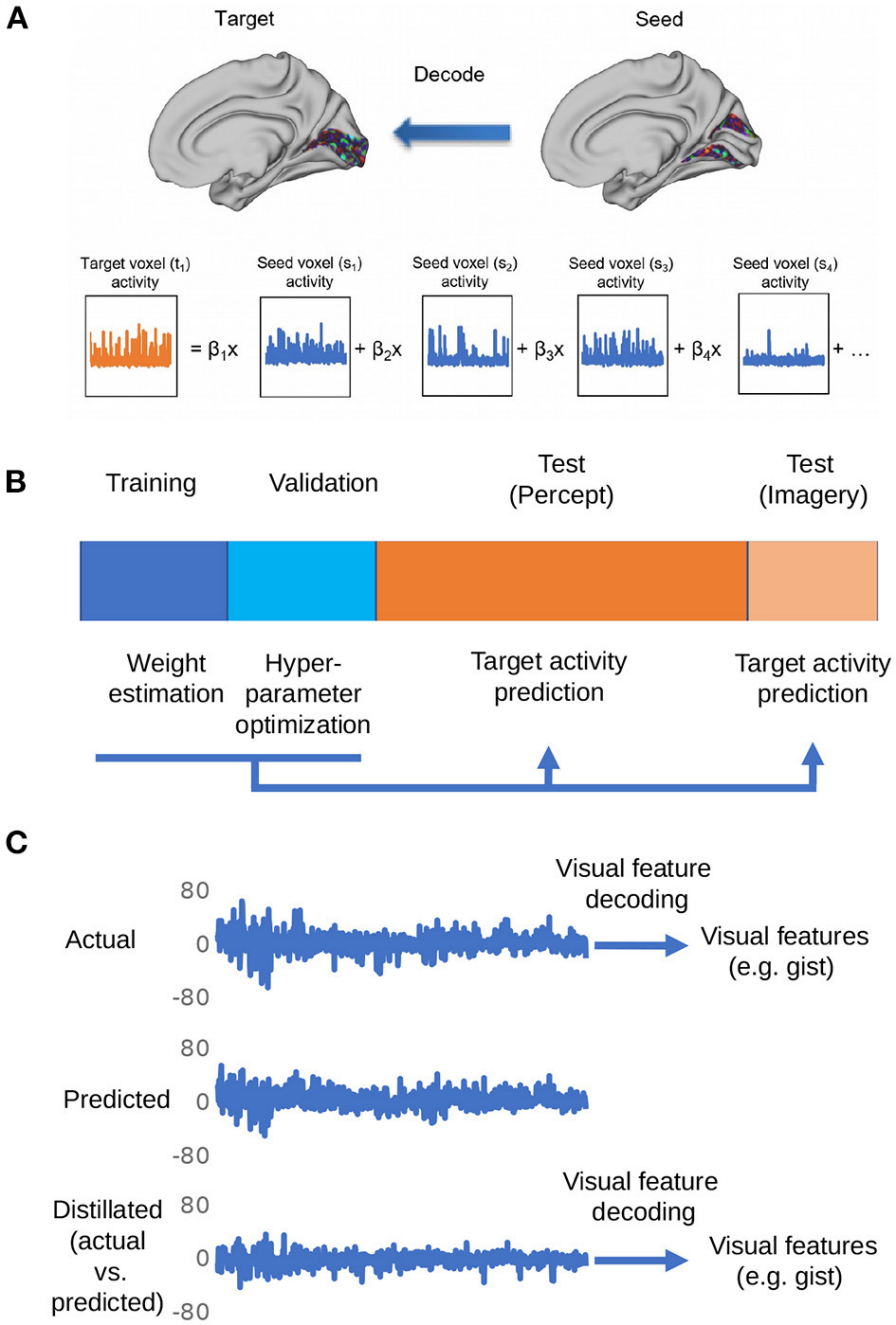
Depiction of the procedures for the proposed distillation analysis. **(A)** Voxel activity in the target region is predicted by a weighted linear summation of all the voxel activities in the seed region. By repeating this procedure for all the voxels in the target region, the whole activity pattern of the target region is predicted. **(B)** The dataset is divided into four subsets: training, validation, perception, and imagery tests. The training data set is used to estimate weights for region-to-region decoding. The validation data set is used for the optimization of hyperparameters (ridge parameter). The weights estimated by the optimization of hyperparameters was used to predict the target region activity in perception and imagery test datasets. **(C)** The representative actual/predicted/distilled activity of a voxel in the perception test data.

Figure 1 shows the overall procedure of our analysis. There are three steps in total: region-to-region prediction, top-down/bottom-up signal elimination, and visual feature predictions.

## 3. Results

### 3.1. Region-to-Region Decoding

**Figure 2:**
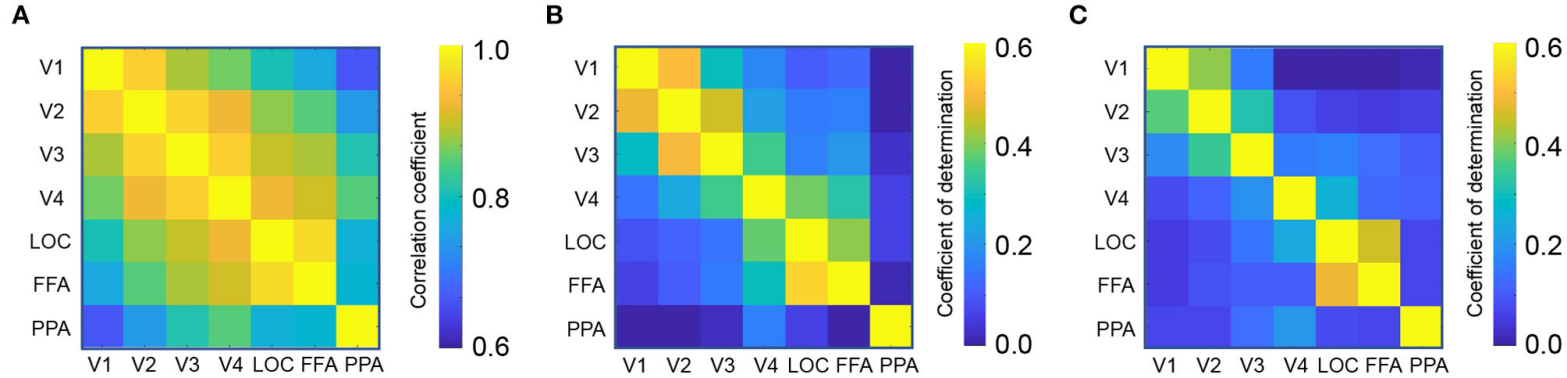
Region-to-region decoding. **(A)** Connectivity matrix between visual object recognition related regions. **(B)** Coefficient of determination in the perception test. **(C)** Coefficient of determination in the imagery test.

First, we calculated the functional connectivity between ROIs associated with the visual processing. Figure 2A shows the connectivity matrix between the ROIs associated with visual object recognition, including the lower visual cortices (V1-V4), the lateral occipital complex (LOC), fusiform face area (FFA), and parahippocampal place area (PPA). The nearby regions, for instance, V1 and V2, exhibited a strong connectivity (Pearson's correlation, mean *r* = 0.96) whereas that between the distant regions such as V1 and PPA, was weaker (*r* = 0.66).

Using each ROI as a seed, a linear ridge regression was performed to predict the activity of all the other ROIs. Figure 1C shows an example of the predicted activity at V1 based on the activity of V2 and its actual activity. We employed the optimal ridge parameter which best predicted the activity in the validation dataset of each seed–target combination for each subject. Similar to the connectivity, the *R*^2^ was high between nearby regions and low between distant regions (Figures 2B,C). Particularly, the *R*^2^ in imagery test was relatively lower than those in perception test, suggesting that the effectiveness of region-to-region decoding may differed according to behavioral task.

### 3.2. Distillation Analysis

The brain activity at a specific region (target region) was subtracted from its predicted activity based on the seed region. Here, the two poles of the visual object recognition were selected, i.e., V1 and FFA. A decoder (Horikawa and Kamitani, 2017) was used to predict the value of the visual features using the multi-voxel fMRI signals of these ROIs. Subsequently, the quality of the prediction was evaluated based on its correlation with the original visual feature.

**Figure 3:**
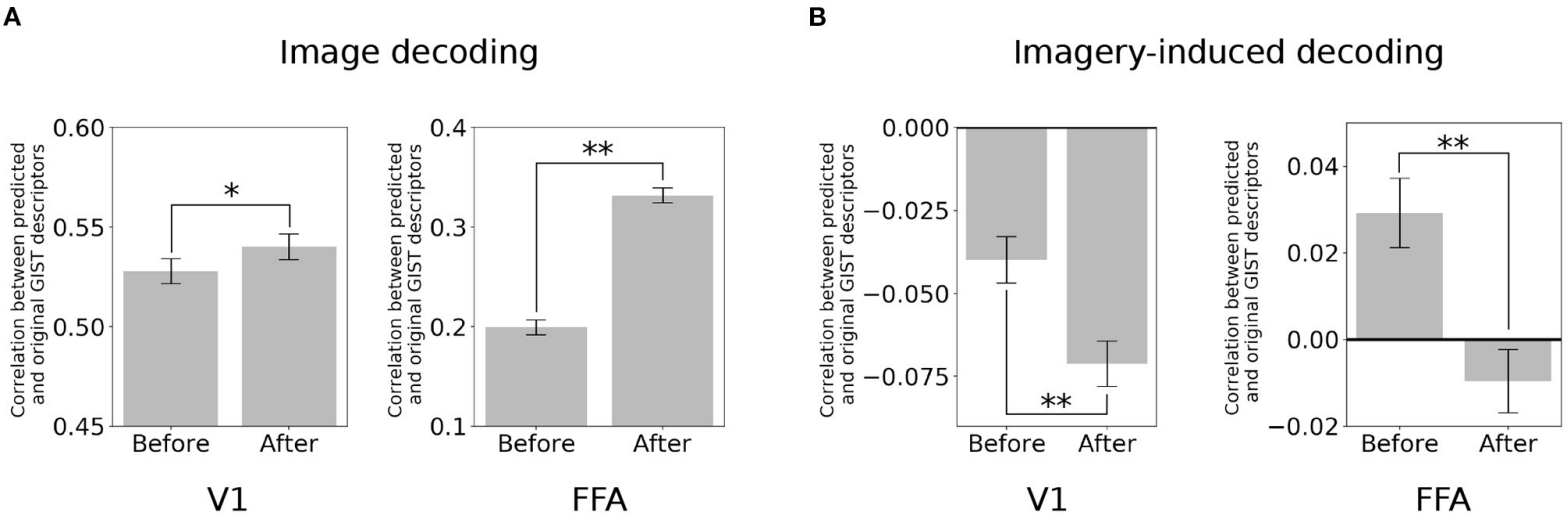
Distillation analysis between V1 and FFA. **(A)** An example of V1 and FFA after distillation in predicting the GIST descriptors. Correlation between predicted and original GIST descriptors in the image-based perception task. **(B)** Correlation between predicted and original GIST descriptors in the class-based imagery task. Error bars represent standard error of measurement (SEM). * uncorrected *p* < 0.05, ** uncorrected *p* < 0.001; two-sided paired *t*-test after Fisher's z-transform; *n* = 500.

In the image perception task, the correlation between the GIST descriptors predicted by the V1 activity and the original signal significantly increased after eliminating the top-down signals from the FFA [Figure 3A; two-sided *t*-test after Fisher's z-transform, *t*_(499)_ = 3.01, *p* < 0.05 [uncorrected]]. Interestingly, a similar increment was also observed in the correlation between the GIST predicted by the FFA and the original after subtracting the bottom-up signals [*t*_(499)_ = 22.67, *p* < 0.001 [uncorrected]]. However, an opposite effect (negative effect) in the imagery task. The correlation between the predicted GIST descriptors and the original one declined at both V1 [*t*_(499)_ = −5.22, *p* < 0.001 [uncorrected]] and FFA [*t*_(499)_ = −6.52, *p* < 0.001 [uncorrected]] after distillation (Figure 3B). These results indicated that during the imagery task, the visual features at these cortices was similar, whereas they were dissimilar during the image perception task.

### 3.3. Effects of Distillation According to the Regional Seed–Target Pair

**Figure 4:**
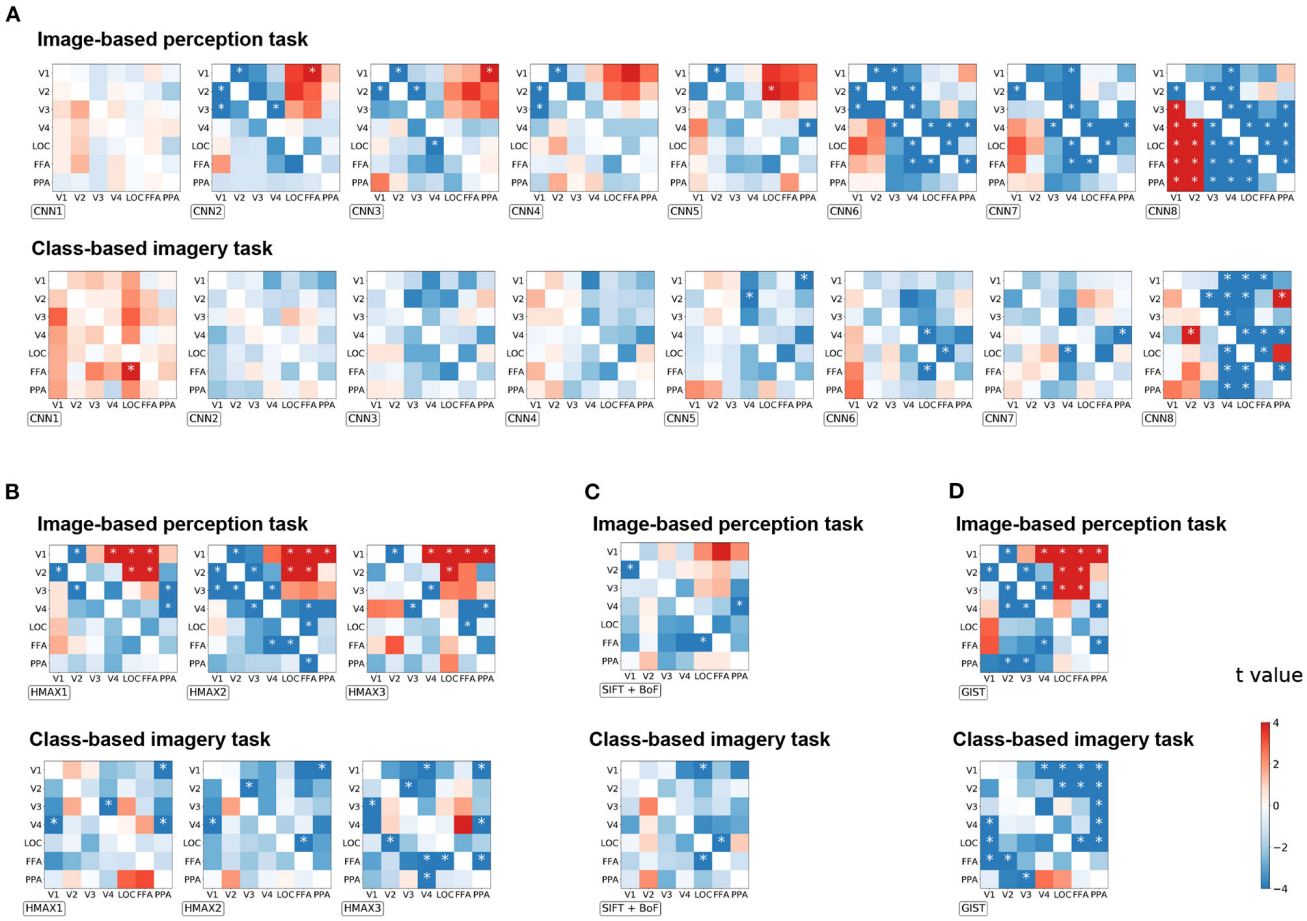
The effect of distillation according to each seed–target pair for image-based perception task and class-based imagery tasks across all groups of visual features. **(A)** CNN visual features, **(B)** HMAX visual features, **(C)** SIFT + BOF, and **(D)** GIST. The difference of the correlation coefficients before and after distillation is arranged into a 7 × 7 matrix for every visual feature. For each group, the matrices are arranged as their complexity increased (from left to right). The diagonals are omitted since they represent the self-distillation which is not within the scope of this study. The horizontal axis represents the seed ROIs, whereas the vertical axis represents the target ROIs. The color bar indicates the t-value of the difference between the corresponding seed–target pair. *p < 0.05 after Bonferroni correction for multiple comparisons (*FWE* < 5%); two-sided paired *t*-test after Fisher's z-transform.

To investigate the effect of distillation in general, all possible seed–target pairs were analyzed. Subsequently, the difference in the correlation coefficient before and after distillation were arranged into a 7 × 7 matrix for every visual feature (Figure 4). The diagonals were omitted since they represent the self-distillation, which is the scope of the current analysis. In this matrix, the upper triangle represents the effect of the top-down signals whereas the lower triangle represents the effect of the bottom-up signals. The positive values indicate that the representations of a visual feature were enhanced by eliminating the modulation of the seed region, and vice versa.

Enhancement of visual feature representations in V1 were observed after eliminating the modulation from FFA for CNN2, HMAX1–HMAX3, and GIST in the image-based perception task (Figure 4). Conversely, the negative effects indicate that the representations of a visual feature were diminished by eliminating the modulation of the seed regions. Such effects were expected to be observed in the combination of brain regions that share the common information. Accordingly, the distillation for the nearby regional pairs caused a negative effect (the blue area) as the nearby regions express strong connectivity as shown in Figure 2A. Interestingly, for complex visual features such as CNN6–8, the negative effects were observed in the pairs of higher visual cortices, such as V4, LOC, FFA, and PPA. These negative effects suggest mutual dependence across the higher visual cortices during the processing of complex visual features.

As expected in the imagery task, almost all pairs had a negative effect, suggesting the existence of a close interaction between the visual cortices during this task (Figure 4B). Specifically, a negative effect was prominent in the upper triangle, indicating that top-down modulation is crucial for visual feature representations during the imagery task. Furthermore, an opposite effect in the imagery task compared to that in the image-based perception task was prominent in CNN8 and GIST.

Since the correlation coefficient is not sensitive to the scaling of data and may be biased under some circumstance (Poldrack et al., 2020), the distillation effect was validated by measuring the MAE (Supplementary Figure 1) and MSE (Supplementary Figure 2). The matrices are arranged in a manner similarly to that of Figure 4. A similar effect of top-down/bottom-up distillation was observed between the MAE/MSE analysis and the correlation analysis. Several exceptions include HMAX3 and GIST visual features in the imagery task. The difference may be due to the fact that the region-to-region decoding in imagery tasks was more difficult, as the coefficient of determinations were lower than those in perception task (Figure 2C).

**Figure 5:**
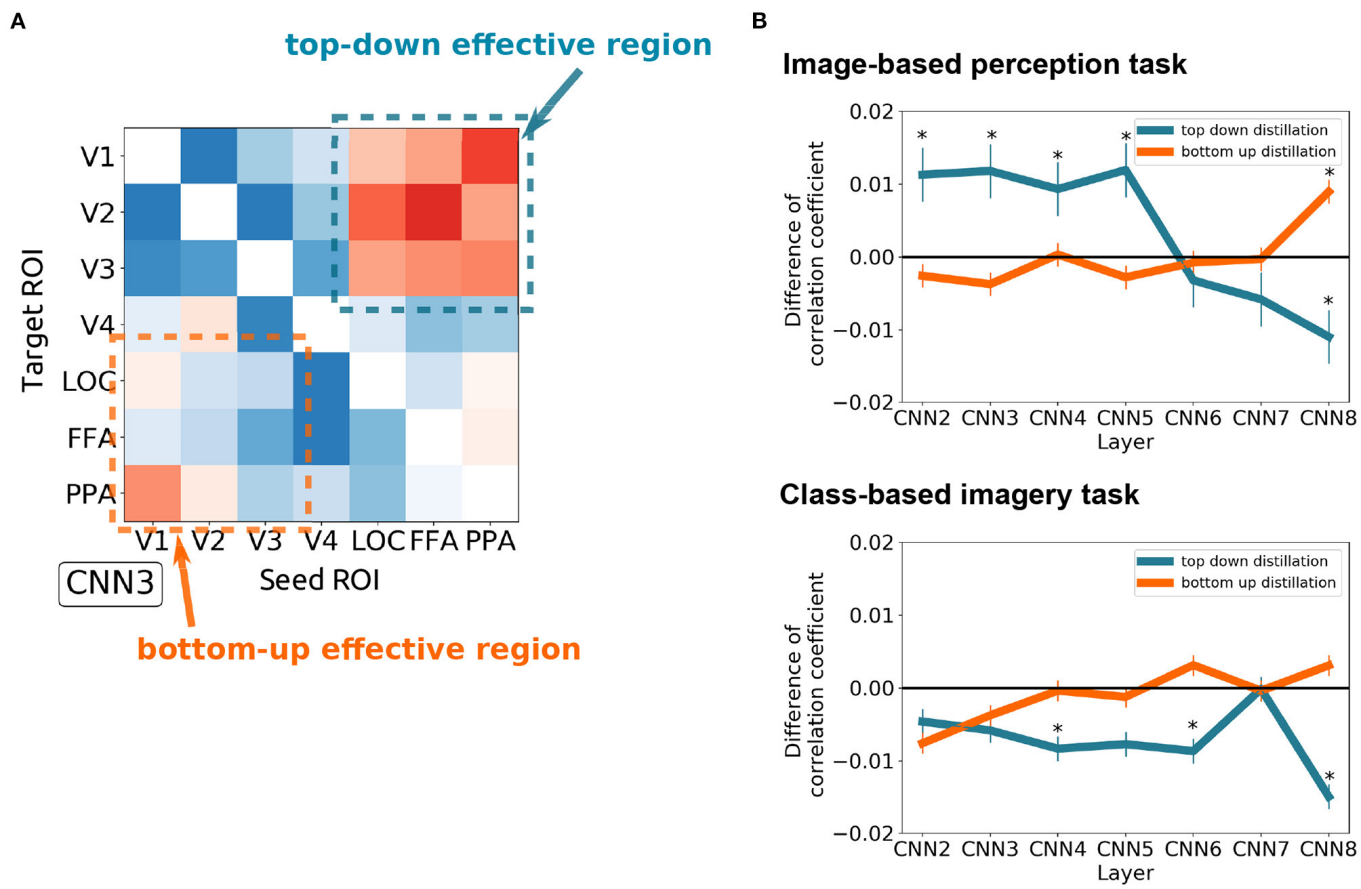
The difference between the top-down distillation and bottom-up distillation. **(A)** An example of the CNN3 's effect matrix illustrating the measured regions. The mean of the 3 × 3 squares at the upper right and lower left of the effect matrices were measured. **(B)** The variance of the two kinds of distillation as the feature complexity increases (from CNN2 to CNN8) in case of image-based perception task (upper) and class-based imagery task (lower). Error bars represent SEM. *p < 0.05 after Bonferroni correction for multiple comparisons (*FWE* < 5%); two sided one sample *t*-test after Fisher's z-transform.

To quantitatively measure the effect of top-down and bottom-up distillation, the mean of the 3 × 3 squares at the upper right and lower left of the effect matrices were measured, respectively (Figure 5A). As the complexity of the features increased, the effect of top-down distillation decreased (Figure 5B), even becoming reversed effect after CNN6. In contrast, the effect of bottom-up distillation slowly increased. This phenomenon was prominent in the case of the image-based perception task. In the case of the class-based imagery task, the predicted features after top-down distillation worsened as the complexity increased.

## 4. Discussion

We have demonstrated a region-to-region decoding technique capable of predicting the neural activity at one region based on the neural activity of another (Figure 2). By eliminating the top-down/bottom-up signals from the original signals (distillation approach), a significant change in the prediction of visual features from brain activity was found. Further analysis revealed three characteristics of the distillation approach.

First, the effectiveness of the distillation approach depends on the connectivity between the regional seed–target pairs. The seed–target pairs that exhibited weak connectivity were more suitable for distillation due to their distinct representations of information. The representations of information were alike between those with strong connectivity, hence, the distillation of top-down/bottom-up signals eliminated their original signals. Second, the distillation approach is also dependent on the type of task, as the imagery task evoked an effect opposed to that of the image-based perception task. Finally, the distillation approach specifies the direction and content of the conveyed information. For example, during an image-based perception task, representations of relatively simpler visual features such as CNN2–5, in V1 were enhanced by eliminating top-down modulations from FFA or PPA, whereas such effects were not observed for CNN6–8. Taken together, the distillation approach is a novel tool for estimating the direction and content of information conveyed across brain regions.

The difference between the visual feature matrices in Figure 4 is expected since the complexity of visual features were found to increase in deeper layers. Previous studies (Horikawa and Kamitani, 2017) synthesized preferred images for each layer using the activation maximization technique, and found the increasing complexity, from simple edge detector representations to complex shapes and textures such as object parts. Conservely, the HMAX model was originally built to mimic the hierarchical processing of the ventral visual pathway. As mentioned in the original paper (Riesenhuber and Poggio, 1999), the HMAX model alternates layers of units by combining simple filters into more complex ones. Finally, SIFT + BoF and GIST are usually considered low-level features designed for object and scene recognition, respectively, as introduced in section 2.

Given our prior knowledge of vision, the FFA is expected to encode the information of facial expressions, and the PPA is expected to encode scene information. Such essential information is undoubtedly delivered from the lower visual cortex (such as V1) via the hierarchical bottom-up processing in the ventral stream. Therefore, they vanished after the bottom-up signals from V1 were distilled (Figures 3, 4). Given a particular case of PPA, the scene information might be removed after distillation of the bottom-up signal, decreasing the decoding performance of the GIST in the PPA. Similarly, in the case of the FFA, the decoding performance of the SIFT + BoF which is related to encoding of facial expressions, decreased. Other information available at FFA and PPA but not directly related to the bottom-up signals, would be maintained. The decoding performance of other information such as GIST, HMAX1–3, and CNN8 increased at FFA. Thus, this result is in line with those of several previous studies in which FFAs were shown to hold information about non-face objects (Haxby et al., 2001; Kanwisher, 2017).

### 4.1. Forward-Backward Interaction Between the Low and High Visual Cortices During Imagery Task

Interestingly, our results show that the information of the visual features was lost after the distillation in the imagery task, suggesting a similarity between the representations of the low-level/high-level regions and their corresponding top-down/bottom-up signals. Due to the lack of concrete “actual” features from visual stimuli, the neural representation of the lower visual cortex aggressively employed the top-down signals. Neural representations at the high-level cortex were reinforced via bottom-up feedback. The quality of such reconstruction depends on the vividness of the imagined object or scene and on the available time for imagery.

Our results are in line with several previous studies (Stokes et al., 2009; Horikawa and Kamitani, 2017; Abdelhack and Kamitani, 2018), which depicts the mental imagery as a type of top-down processing. Previous studies reported the common neural representations during both perception and mental imagery (Albers et al., 2009, 2013; Stokes et al., 2009; Reddy et al., 2010; Cichy et al., 2012; Xing et al., 2013; Johnson and Johnson, 2014; Naselaris et al., 2015; Horikawa and Kamitani, 2017). Our analysis revealed the different compositions of these neural representations concerning their utilization of top-down/bottom-up signals.

### 4.2. Significance and Limitations of the Distillation Analysis

Whereas the functional connectivity and effective connectivity analyses focus on estimating the strength of regional connectivity, the distillation approach specifies the content of the conveyed information between regions as well as interregional connectivity. Figure 5 demonstrates that the top-down modulation during the visual perception task could diminish the simpler visual features (i.e., CNN2–5) represented in the early visual cortices (i.e., V1–V3). With the distillation approach, the effect of top-down modulation can be analytically eliminated, resulting in the enhancement of simpler visual feature representations in the early visual cortices. It should be noted that the effects of distillation were not observed for any visual features. Rather, these effects were specific to the visual features of interest as well as the combination of the seed and target.

The current approach has two limitations. First, the distillation results can be artificial if the actual connectivity between the seed and target does not exist. Hence, results of the distillation analysis should be interpreted with caution to avoid misinterpretation. *In this study, both the correlation coefficient and the additional MAE/MSE were used as the metrics to evaluate the distillation results. A result was considered reliable if it was consistent across these metrics, i.e., the effect of distillation in perception task, and across CNN visual feature in imagery task*. The current results were derived from our prior assumptions, wherein the prediction of a low-level ROI based on the activity of a high-level region represents its top-down signals from that specific region, and vice versa. Second, the recurrent effect was not considered (i.e., the effect caused by the circulation of information, from FFA to V1 to FFA), because its complexity may cause artifacts. Furthermore, we assume that the recurrent effect would be relatively smaller than the direct modulation at the moment of observation and, thus, could be negligible. To specify the precise modulatory effect between regions (e.g., from V1 to FFA), the recurrent effect should be included in the future model.

### 4.3. Potential Uses of the Distillation Analysis

The development of brain-computer interfaces (BCIs) will benefit from our distillation approach for precise decoding. Incorporating with the now mature image reconstruction by deep learning (Shen et al., 2019), our approach may reconstruct what people see even though it is not visually recognizable. One could also consider repeating the distillation analysis to obtain a better representation of the information at an ROI. Furthermore, the distillation analysis is applicable to the decoding of other sensory modalities, such as auditory and haptics feedback. Cross-distillation between different modalities would help us to gain better insights into their intervention in future work.

## Data Availability Statement

Publicly available datasets were analyzed in this study. This data can be found here: https://github.com/KamitaniLab/GenericObjectDecoding.

## Ethics Statement

The studies involving human participants were reviewed and approved by the Ethical Committee of the National Institute for Physiological Sciences of Japan. The patients/participants provided their written informed consent to participate in this study.

## Author Contributions

NS and JC contributed to the design and provided the conception and overall guidance for the project. SN, TP, and JC contributed to the data analysis and interpretation. TP and JC contributed to the initial drafting of the manuscript. All authors contributed to the writing, revision, and approval of the manuscript.

## Funding

This work was supported by a grant from Japan Society for the Promotion of Science (JSPS) KAKENHI to TP (Grant Number 19K20390), JSPS KAKENHI to JC (Grant Number 21H02806, 18H05017, and 17H06033), and a grant from Japan Agency for Medical Research and Development (AMED) to JC (Grant Number JP21dm0207086) and NS (JP18dm0107152 and JP20dm0307005).

## Conflict of Interest

JC and SN are employed by Araya Inc. The remaining authors declare that the research was conducted in the absence of any commercial or financial relationships that could be construed as a potential conflict of interest.

## Publisher's Note

All claims expressed in this article are solely those of the authors and do not necessarily represent those of their affiliated organizations, or those of the publisher, the editors and the reviewers. Any product that may be evaluated in this article, or claim that may be made by its manufacturer, is not guaranteed or endorsed by the publisher.
