# Supplementary material for: Distillation of Regional Activity Reveals Hidden Content of Neural Information in Visual Processing
Source: Front Hum Neurosci. 2021 Nov 26;15:777464. doi: 10.3389/fnhum.2021.777464 (PMC8664645; doi:10.3389/fnhum.2021.777464)
Supplement: Supplementary file 1 [file Data_Sheet_1.PDF]

# Supplementary Material

## 1 SUPPLEMENTARY TABLES AND FIGURES

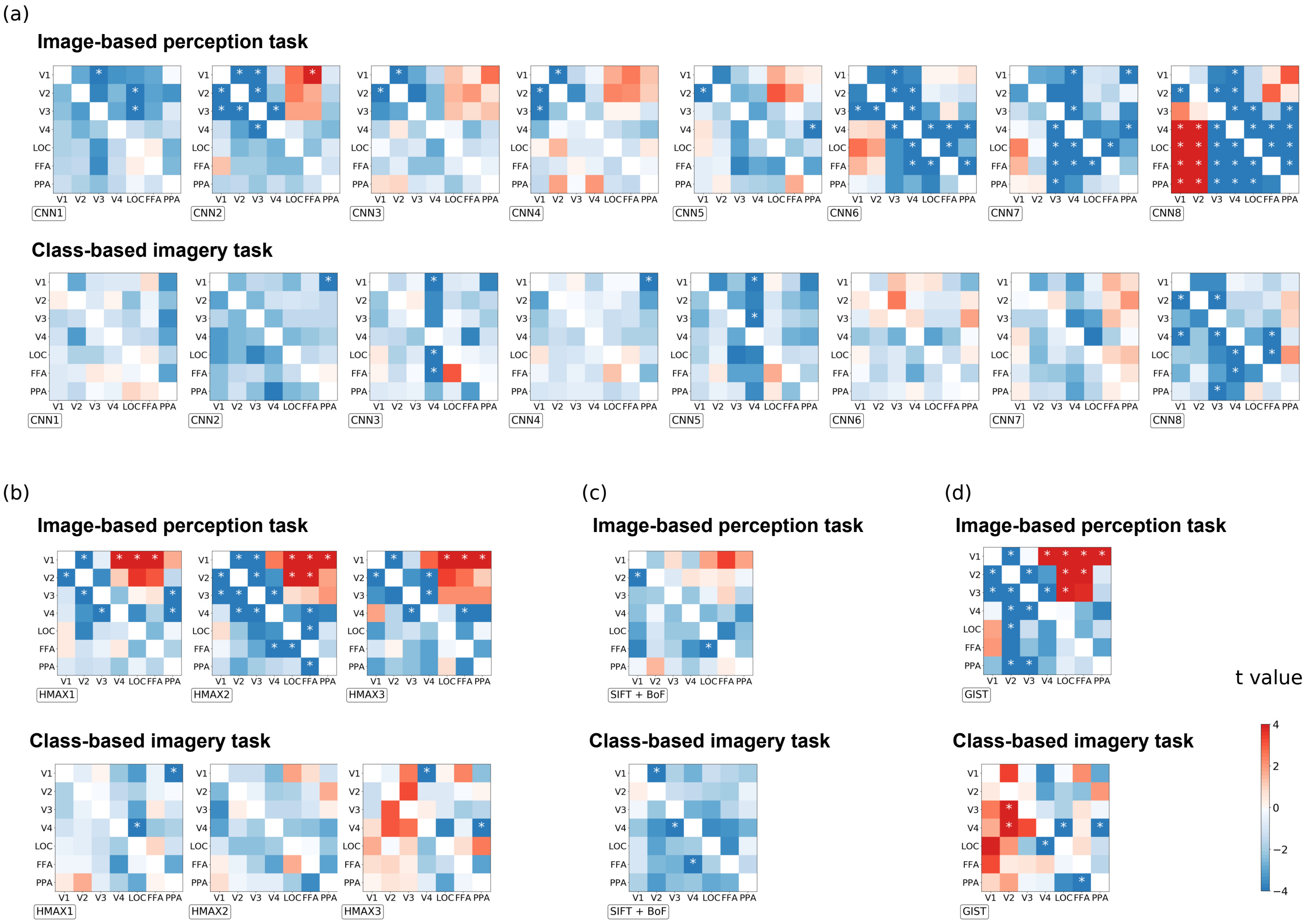


**Figure S1.** The effect of distillation measured by mean absolute error (MAE) across all groups of visual features. (a) CNN visual features, (b) HMAX visual features, (c) SIFT + BOF, (d) GIST. The difference of the MAE before and after distillation is arranged into a $7 \times 7$ matrix for every visual feature. For each group, the matrices are arranged as their complexity increased (from left to right). The diagonals are omitted since they represent the self-distillation which is not within the scope of this study. The horizontal axis represents the seed ROIs, whereas the vertical axis represents the target ROIs. The color bar indicates the t-value of the difference between the corresponding seed–target pair. $*p < 0.05$ after Bonferroni correction for multiple comparisons ($FWE < 5\%$); two-sided paired t-test after Fisher's z-transform.

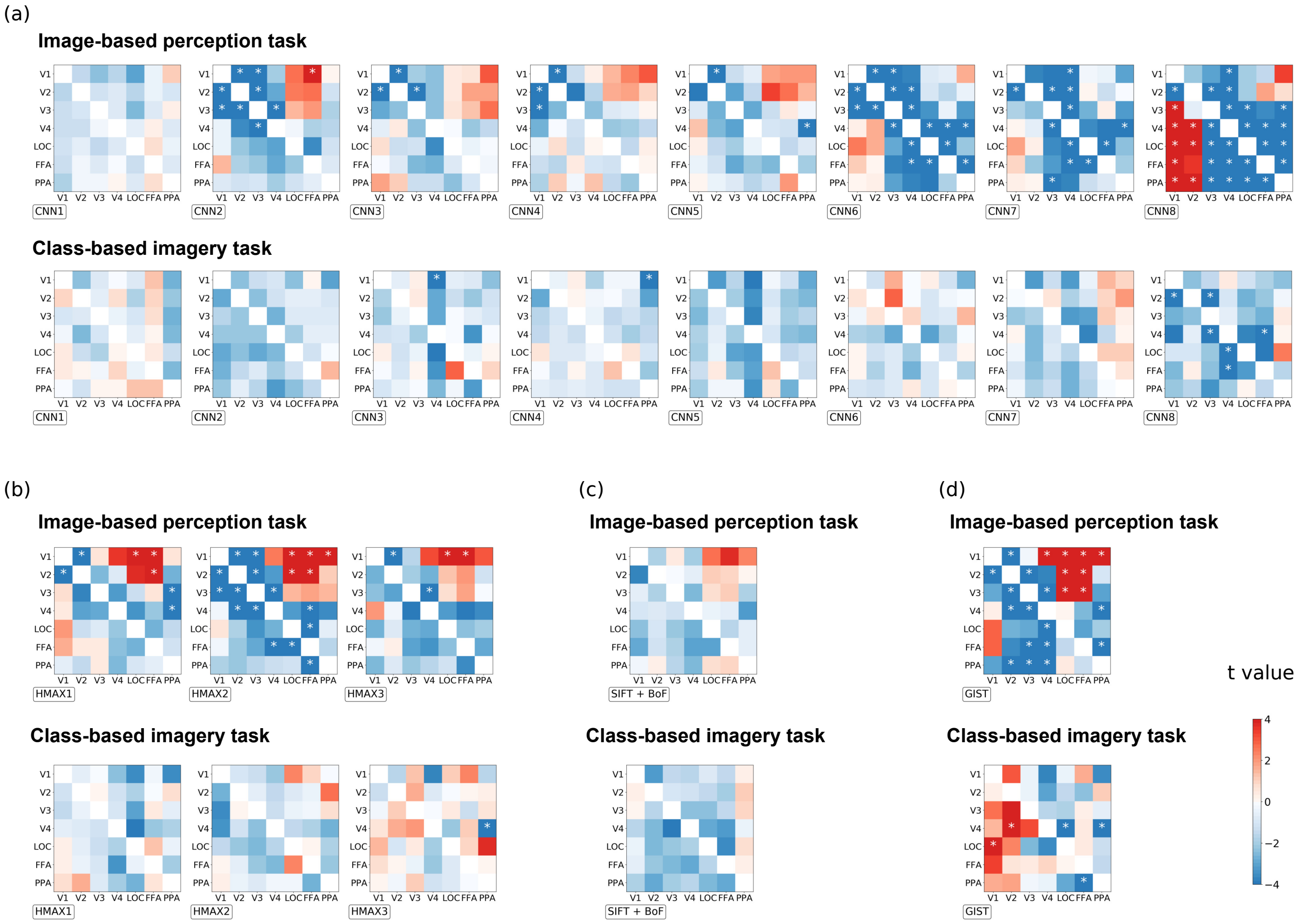


**Figure S2.** The effect of distillation measured by mean squared error (MSE) across all groups of visual features. (a) CNN visual features, (b) HMAX visual features, (c) SIFT + BOF, (d) GIST. The difference of the MSE before and after distillation is arranged into a $7 \times 7$ matrix for every visual feature. For each group, the matrices are arranged as their complexity increased (from left to right). The diagonals are omitted since they represent the self-distillation which is not within the scope of this study. The horizontal axis represents the seed ROIs, whereas the vertical axis represents the target ROIs. The color bar indicates the t-value of the difference between the corresponding seed–target pair. $*p < 0.05$ after Bonferroni correction for multiple comparisons ($FWE < 5\%$); two-sided paired t-test after Fisher's z-transform.
